# Design of a Custom RT-qPCR Array for Assignment of Abiotic Stress Tolerance in Traditional Portuguese Grapevine Varieties

**DOI:** 10.3389/fpls.2017.01835

**Published:** 2017-10-25

**Authors:** Luísa C. Carvalho, Marília Silva, João L. Coito, Margarida P. Rocheta, Sara Amâncio

**Affiliations:** Linking Landscape, Environment, Agriculture and Food, Instituto Superior de Agronomia, Universidade de Lisboa, Lisbon, Portugal

**Keywords:** abiotic stress, acclimation, custom array, grapevine, qPCR, stress tolerance, transcriptomics

## Abstract

Widespread agricultural losses attributed to drought, often combined with high temperatures, frequently occur in the field, namely in Mediterranean climate areas, where the existing scenarios for climate change indicate an increase in the frequency of heat waves and severe drought events in summer. Grapevine (*Vitis vinifera* L.) is the most cultivated fruit species in the world and the most valuable one and is a traditional Mediterranean species. Currently, viticulture must adjust to impending climate changes that are already pushing vine-growers toward the use of ancient and resilient varieties. Portugal is very rich in grapevine biodiversity, however, currently, 90% of the total producing area is planted with only 16 varieties. There is a pressing need to understand the existing genetic diversity and the physiological potential of the varieties/genotypes available to be able to respond to climate changes. With the above scenario in mind, an assembly of 65 differentially expresses genes (DEGs) previously identified as responsive to abiotic stresses in two well studied genotypes, ‘Touriga Nacional’ and ‘Trincadeira,’ was designed to scan the gene expression of leaf samples from 10 traditional Portuguese varieties growing in two regions with distinct environmental conditions. Forty-five of those DEGs proved to be associated to “abiotic stress” and were chosen to build a custom qPCR array to identify uncharacterized genotypes as sensitive or tolerant to abiotic stress. According to the experimental set-up behind the array design these DEGs can also be used as indicators of the main abiotic stress that the plant is subjected and responding to (drought, heat, or excess light).

## Introduction

Grapevine (*Vitis vinifera* L.) is the most cultivated fruit crop in the world and the most valuable one. It has been cultivated for *circa* 8000 years and is the only Mediterranean/Western Asiatic representative of the genus *Vitis*, having been domesticated from its wild ancestor *V. vinifera* spp *sylvestris*. The enormous genetic diversity found in this species derives most likely from the combination of several processes. The foremost is the existence of numerous domestication events from local wild *Vitis sylvestris* vines (Arroyo-Garcia et al., 2006), followed by subsequent crosses between already domesticated and still wild local vines, the ancient practice of growing seedlings from spontaneous crosses and, finally, conventional breeding (Pelsy, 2010).

Grapevine varieties are well adjusted to a wide range of climates and in 2015 production covered *circa* seven and a half million hectares and more than 75.7 million tons of berries were produced worldwide (OIV, 2016). Nowadays, most wine producing countries use a small number of varieties in a large percentage of their total vineyard area. This recurrent practice leads to the marginal cultivation or even the extinction of a vast number of traditional and local varieties.

However, the worldwide demand for high quality wines is growing, reviving the interest to use traditional cultivars to craft products of excellence and to perform exhaustive biochemical characterizations of the organoleptic traits they possess (Venturini et al., 2013). This necessity, together with the pressing need of viticulture to adjust to impending climate changes already taking place in traditional grape-growing areas, such as the Mediterranean basin, is pushing vine-growers toward the use of ancient and more resilient varieties and also toward a use of a combination of varieties and/or genotypes instead of only one. Therefore, growers need to understand the genetic diversity and the physiological potential of the varieties/genotypes available, in order to exploit their capacity to adjust to abiotic stresses while still producing good quality grapes.

Portugal is very rich in grapevine biodiversity, with 1,482 different varieties in use in the XIX century. However, nowadays there are 341 officially authorized varieties for wine production, with 90% of the total producing area planted with 13 red varieties and 3 white varieties (Almandanim et al., 2007). Such an irreversible loss of diversity dangerously shrinks the genetic pool, increasing the crop’s susceptibility to climate changes and new diseases and pests. The *ex situ* field collections have a crucial role in germplasm conservation. Representatives of all Portuguese grapevine varieties are preserved in the Portuguese Grapevine Collection established at Estação Vitivinícola Nacional (Dois Portos) and in the Experimental Centre for Conservation of Grapevine Diversity of the Portuguese Association for Grapevine Diversity (PORVID) in Pegões.

Arabidopsis genome sequencing (Kaul et al., 2000) marked a turning point in genomics research, namely in the effects of different abiotic stresses at gene expression level. To quote the most cited reports, the model plant was used in studies addressing water, heat, cold, salt and biotic stresses, individually and in pairs (Seki et al., 2002; Taji et al., 2002; Narusaka et al., 2004; Rizhsky et al., 2004; Busch et al., 2005; Sakuma et al., 2006; Kilian et al., 2007) in controlled experimental conditions. As other plant genomes were sequenced, production purposes drove the genomic research to economically important crops, such as grapevine (Jaillon et al., 2007; Velasco et al., 2007), but maintaining the experimental controlled set-ups (Cramer et al., 2007; Grimplet et al., 2007; Deluc et al., 2009; Carvalho et al., 2011; Venturini et al., 2013; Rocheta et al., 2014). However, following research that addressed normal and pathological situations in humans in the early 2000s (Lewohl et al., 2000; Sallinen et al., 2000; Xu et al., 2000), plant research adopted systemic approaches, in field growth conditions (Zenoni et al., 2010; Fasoli et al., 2012; Dal Santo et al., 2013; Venturini et al., 2013; Nwafor et al., 2014). In fact, in the field, plants meet simultaneously the effects of various stresses (Fujita et al., 2006; Mittler, 2006; Mittler and Blumwald, 2010), which are normally different from the sum of the effects induced by individual stress factors (Atkinson and Urwin, 2012). Recently, a study at the transcriptional, posttranscriptional and translational levels in grapevine subjected to heat stress was undertaken and showed that the regulation of several key heat shock proteins and transcription factors occur at those three levels, depending on the intensity of the stress applied (Jiang et al., 2017). Considering the effects of abiotic stresses, they actually act in tandem, heat stress in association with water deficit, eventually also with high radiation, water shortage plus salt stress or low temperatures together with high radiation, as depicted by Mittler (2006) in the *Stress Matrix*. While the application of individual abiotic stresses in controlled conditions points out their acute effects, the combination of stresses gives information about the synergies or antagonisms between the effects of those stresses but field conditions, on the other hand, allow to obtain the actual physiological and genomic signature of the natural stress conditions.

In previous works, we have thoroughly characterized the different and contrasting leaf responses of the grapevine varieties ‘Touriga Nacional’ (TN) and ‘Trincadeira’ (TR) to single and combined water, heat, and light stresses in controlled conditions. Field plants under water deficit were also monitored, and results of different physiological parameters and transcriptomic data obtained with the GrapeGen Affymetrix GeneChip^®^ were integrated. This approach identified several stress responsive transcripts (Rocheta et al., 2016) and allowed to classify TN as a variety that can withstand severe levels of stress, without its physiology being much affected while TR is more sensitive (Carvalho et al., 2015, 2016).

Here, we aimed to assign the tolerance to abiotic stress of a group of economically relevant traditional grapevine Portuguese varieties, three red and seven white, five of which are empirically considered sensitive and the other five tolerant. To obtain a broad picture of the responses of each variety to abiotic stress, samples were obtained in vines from two regions with distinct environmental conditions. To scan the expression of leaf samples of those varieties we designed a custom qPCR array of 65 differentially expressed genes (DEGs) previously identified in TN and TR and assembled the obtained transcriptomic data. We propose that a number of those DEGs (forty five, plus three reference genes) can be associated to “abiotic stress” and used as indicators of the main abiotic stress that the plant is subjected and responding to (drought, heat, or excess light) and thus incorporated in a custom array to characterize the level of tolerance to stress of most uncharacterized genotypes. As a result, the integration of a transcript profiling platform with physiological parameters and economically relevant traits can be achieved.

## Materials and Methods

### The Control Varieties and Stress Treatments Applied

Well characterized plants of the grapevine varieties ‘TN’ (tolerant to stress) and ‘TR’ (sensitive to stress) were cultivated under abiotic stress in controlled and field conditions, as described (Supplementary Table S1; Rocheta et al., 2016). In both varieties samples were taken simultaneously when the pre-dawn leaf water potential (Ψw, quantified with a pressure chamber, Model 600, PMS Instruments Company, Albany, OR, United States) was *circa* -0.7 MPa in the NI treatment and higher than -0.2 MPa in the FI treatment. Samples comprised the first and second totally expanded leaves, per plant and per treatment, which were frozen in liquid nitrogen until RNA extraction. Pruned wood of the above described plants of TN and TR provided the cuttings that were grown in growth room under controlled conditions as described in Carvalho et al. (2016) and Supplementary Table S1. Samples consisted of the first, second, and third totally expanded leaves counting from the shoot apex and were taken at the end of LS and HS treatments and, for WS, when Ψw reached the chosen value. Leaves were frozen in liquid nitrogen and stored at -80°C until RNA extraction.

### The Traditional Varieties Used and Field Conditions in Both Locations

Ten varieties, most of them native to Portugal, were chosen to assess their response to stress and to choose the transcripts to include in the custom stress array. ‘Antão Vaz’ (AV) is a native white variety, gown mostly in Alentejo, and is sensitive to drought (IVV, 2011); ‘Bastardo’ (BA; syn ‘Trousseau’), a red variety originated from France but already showing high genetic variability in Douro and Dão, an indication that it has been cultivated there for a long time (Martins and Gonçalves, personal communication), its levels of tolerance to abiotic stress haven’t been studied; ‘Castelão’ (CT), the most important red variety in the wine area where Pegões is located (Peninsula de Setúbal), it is the commonly used reference for phenological stages in red varieties in Portugal (IVV, 2011), its genotypes show various levels of tolerance to stress; ‘Cerceal Branco’ or ‘Cerceal do Douro’ (CB), a white variety traditionally cultivated in Douro and Dão, is considered tolerant to drought (IVV, 2011); ‘Encruzado’ (EN), traditionally grown in Dão, is a white variety known for its tolerance to drought (IVV, 2011); ‘Fernão Pires’ (FP), a white variety originated in Bairrada but also grown in Ribatejo and Estremadura, is the commonly used reference for phenological stages in white varieties in Portugal and is sensitive to drought (IVV, 2011); ‘Moscatel Graúdo’ or ‘Moscatel de Setúbal’ (MG; syn ‘Muscat Alexandria’), a white variety originated in the eastern Mediterranean but grown in Peninsula de Setúbal for long, where it already has acquired high genetic variability (Martins and Gonçalves, personal communication), it is considered tolerant to drought (IVV, 2011); ‘Tinta Barroca’ (TB) and ‘Touriga Franca’ (TF), two red varieties traditional in Douro, resulting from crossings between ‘Marufo’ and TN, TB is sensitive to excess heat and drought (IVV, 2011) while TF is tolerant to abiotic stress; and finally, ‘Viosinho’ (VZ), a white variety originated in Douro, described as tolerant to heat (IVV, 2011).

**FIGURE 1:**
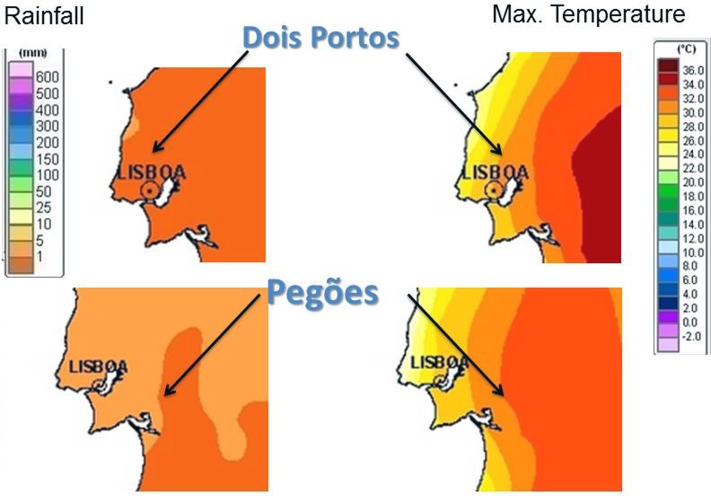
Weather conditions (maximum temperatures and rainfall) in Dois Portos and Pegões when sampling took place.

The 10 varieties used for the experiment were grown in Estação Vitivinícola Nacional in Dois Portos and in Experimental Centre for Conservation of Grapevine Diversity of the Portuguese Association for Grapevine Diversity (PORVID) in Pegões, in the conditions described in Supplementary Table S1. Climate conditions in the two regions and seasons are shown in **Figure 1** (data retrieved from IPMA^1^). Samples were taken simultaneously in all varieties when the Ψw was -0.7 MPa (global average, no significant differences found between varieties, *p*-value = 0.496 for Kruskal–Wallis chi-squared test). Samples consisted of the first, second, and third totally expanded leaves from three plants per variety (three biological replicates), which were frozen in liquid nitrogen and stored at -80°C until RNA extraction.

### RNA Extraction

Samples were ground in the presence of liquid nitrogen with a mortar and pestle. Total RNA was extracted with the Spectrum^TM^ Plant Total RNA kit (Sigma-Aldrich, St. Louis, MO, United States). In all samples nucleic acid concentration was quantified by spectrophotometry using the software Gen5 1.09 (Synergy HT, Bio-Tek Instruments, Winooski, VT, United States). The quality of the extracted RNA was evaluated using the A260/A280 and A260/A230. To be used, samples had to have ratios A260/A280 between 1.8 and 2.1 and A260/A230 between 2.0 and 2.2. Total RNA integrity was assessed through 1% agarose gel electrophoresis under denaturing conditions.

### cDNA Synthesis for qPCR

RNA samples were treated with RQ1 RNase-Free DNase (Promega, Madison, WI, United States). cDNA was synthesized from 2 μg of total RNA using oligo(dT)_20_ in a 20 μL-reaction volume using RevertAid Reverse Transcriptase (Fermentas Life Science, Helsingborg, Sweden) according to the manufacturer’s recommendations. cDNA was tested for gDNA contamination in PCRs using intron spanning primers that yield a 229 bp amplicon in cDNA and a 547 amplicon in gDNA. Amplicon sizes were compared in 2% agarose gels together with the molecular weight marker 1Kb+ (Invitrogen) and no gDNA contamination was detected. cDNA was stored at -20°C until further use.

### qPCR

Primers were designed using the software Beacon Designer (Premier Biosoft) using a primer length of 20 ± 2 bp, melting temperature of 60°C ± 2°C, a guanine–cytosine content of *circa* 50% and an expected amplicon size of 180–280 bp. Sequences were identified in a previous experiment of stress using the 23K custom-made Affymetrix Vitis GeneChip applied to the varieties TN and TR (Rocheta et al., 2016; accession: GSE57669). Real-time qPCR reactions were performed in 96 well white reaction plates (Bio-Rad, Hercules, CA, United States), using an IQ5 Real Time PCR (Bio-Rad, Hercules, CA, United States) with three biological replicates and two technical replicates. The 20 μL reaction mixture was composed of 50-fold diluted cDNA (1 μL), 0.5 μM of each gene-specific primer and 10 μL master mix (SsoFast_EvaGreen Supermix, Bio-Rad, Hercules, CA, United States). Amplification of PCR products was monitored through the intercalation of Eva-Green (included in the master mix). The program applied was the following: initial polymerase activation, 95°C, 3 min; then 40 cycles at 94°C 10 s (denaturation), 60°C 20 s (annealing), 72°C 15 s (extension). After cycling was completed, a melting curve analysis to confirm the correct amplification of target gene fragments and the lack of primer dimmers was done. The PCR products were run on 2% agarose gels to make sure that there was only one amplicon of the expected size. PCRs with each primer pair were also performed on samples lacking cDNA template, in triplicate (no template controls). To assess amplification efficiency of the candidate genes, identical volumes of cDNA samples were diluted and used to generate five-point standard curves based on a five-fold dilution series (1; 1:5; 1:25; 1:125; 1:625), in triplicate. Amplification efficiency (*E*) is calculated as *E* = 10(-1/*a*)-1, “*a*” being the slope of the linear regression curve [*y* = *a* log(*x*) + *b*] fitted over the log-transformed data of the input cDNA dilution (*y*) plotted against the respective quantification cycle (*C*q) values (*x*). *E*-values of the target genes were considered comparable when they did not exceed 100 ± 10%, corresponding to a standard curve slope of 3.3 ± 0.33.

To generate a baseline-subtracted plot of the logarithmic increase in fluorescence signal (ΔRn) versus cycle number, baseline data were collected between cycles 5 and 17. All amplification plots were analyzed with an Rn threshold of 0.2 at the beginning of the region of exponential amplification, to obtain *C*q (quantification cycle) and the data obtained were exported into a MS Excel workbook (Microsoft Inc., United States) for analysis. Reference genes used were ACT, TIF, and TIF-GTP (Coito et al., 2012).

### Statistical Analysis

For the relation between the expressions of the selected genes and the reference genes the relative quantity values were transformed into log_2_ (thus rendering them parametric) and tested through ANOVA. When the *p*-value of the ANOVA was lower than 0.05 a Tukey test was performed and statistically significant differences were accepted for a *p*-value lower than 0.05. ANOVAS and the respective *post hoc* tests were made in R (version 2.15.1, Copyright 2012; The R Foundation for Statistical Computing).

## Results

### Description of the Chosen DEGs

A previous experiment using individual abiotic stresses in controlled conditions (drought, heat, and excess light) was applied to plants of the varieties TN and TR. Leaf samples from those plants were used for microarray analysis (Rocheta et al., 2016). Fully irrigated and non-irrigated field plants grown in the Experimental Centre for Conservation of Grapevine Diversity in Pegões were also subjected to microarray analysis (Rocheta et al., 2016). For each variety, the five DEGs that were most upregulated and downregulated in the microarray experiment in TN and in TR in each stress situation were selected as markers of the respective stress treatment. In total, 65 DEGs were chosen, 32 that were upregulated in the array and 33 that were downregulated, some being markers of more than one stress treatment. This selection comprised several genes without annotation (Unknown category in GrapegenDB and MapMan) as long as homologous transcripts were identified at NCBI database^2^. These 65 DEGs, together with three reference genes, make up the custom stress array tested here.

**FIGURE 2:**
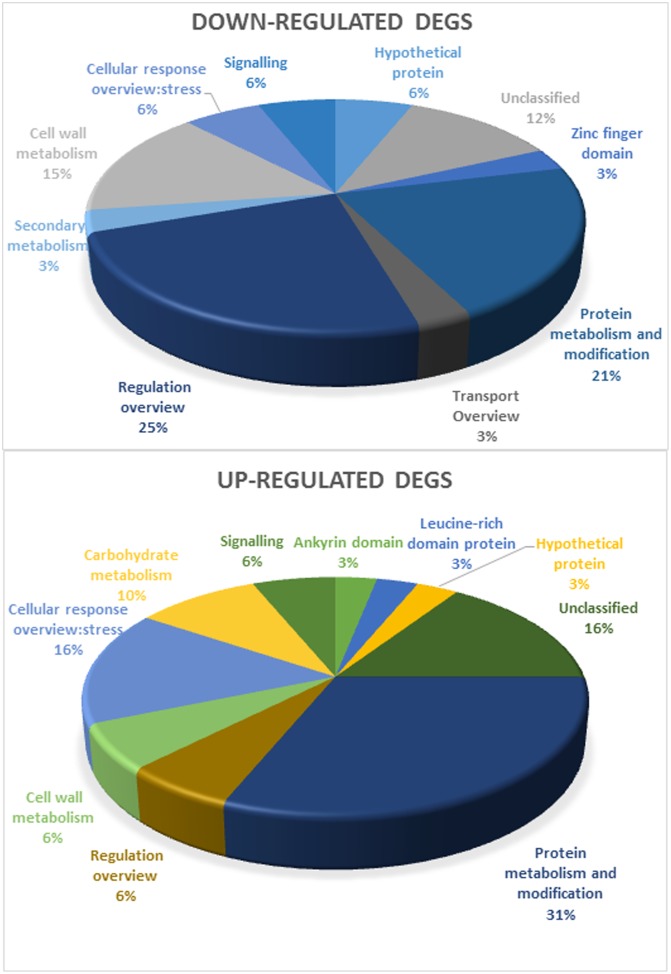
Functional categorization of annotated genes (separated in downregulated and upregulated DEGs in Touriga Nacional and Trincadeira) by MapMan analysis.

The DEGs used in this study are represented in Supplementary Table S2, based on functional categories (Grapegen Functional Categories and MapMan, Thimm et al., 2004). Unexpectedly, DEGs included only a few effector proteins typically associated with the response to drought, such as proteins with chaperone activity (dehydrins and LEA proteins) (Umezawa et al., 2006; Shao et al., 2008), all upregulated in response to drought in the field and in controlled conditions. The few proteins associated with cell wall remodeling (Tenhaken, 2014), also important in the response to drought, were mostly downregulated. DEGs also included regulatory components of known stress-response pathways, such as HSPs, upregulated in the field and in controlled heat stress and downregulated in controlled light stress. All but two TFs and signaling elements were downregulated, in light and heat stress conditions. Protein metabolism and modification and Response to stress make up for the largest of upregulated bins (**Figure 2**) while regulation overview and protein metabolism were the most downregulated bins. The bin secondary metabolism only comprised downregulated DEGs while carbohydrate metabolism only showed upregulated DEGs. All this suggests a tight control of stress responses at both the transcriptional and signaling levels.

### Analysis of the qPCR Array

**FIGURE 3:**
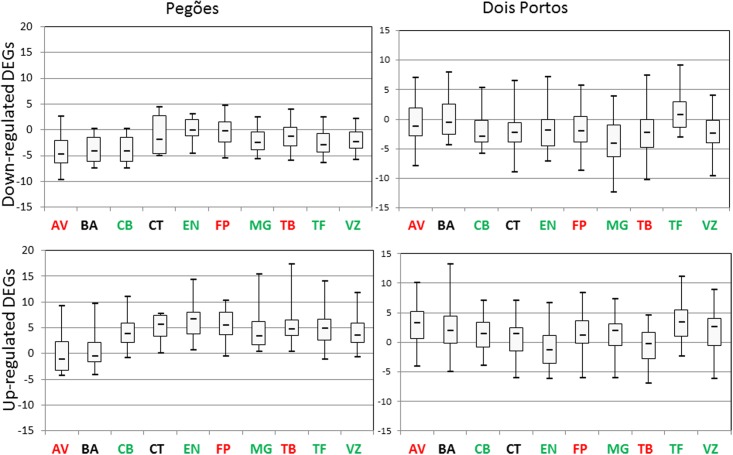
Box-plots representing the distribution of the expression of upregulated and downregulated DEGs [log_2(*gene expression ratio*)_] in the grapevine varieties studied in both locations (Dois Portos and Pegões). The color-code of the varieties’ names, according to the empirical classification, is the following: red: sensitive, green: tolerant, black: unknown.

In both environmental conditions, the genotypes studied were evaluated for the expression of the chosen DEGs. In **Figure 3**, the distribution of expression of downregulated and upregulated DEGs is represented by whisker-box plots, for all varieties and in the two locations. The empirical assumption of tolerance/sensitivity is indicated by the variety’s abbreviation in green (tolerant), red (sensitive), or black (unknown). It is possible to verify that the stability of expression of downregulated genes was higher than that of upregulated genes and that in Pegões the stability was also higher than in Dois Portos. It is also possible to verify that the majority of downregulated genes were consistent with their expected regulation tendency in most varieties while upregulated genes changed expression tendency, for example in Pegões, CT, EN, and FP many downregulated genes were upregulated and in Dois Portos many upregulated genes were downregulated in EN and TB, as the respective distribution of boxes/whiskers indicate. This global gene analysis did not find any pattern of expression related to tolerance.

### Relative Relevance of Functional Categories in the Varieties

**FIGURE 4:**
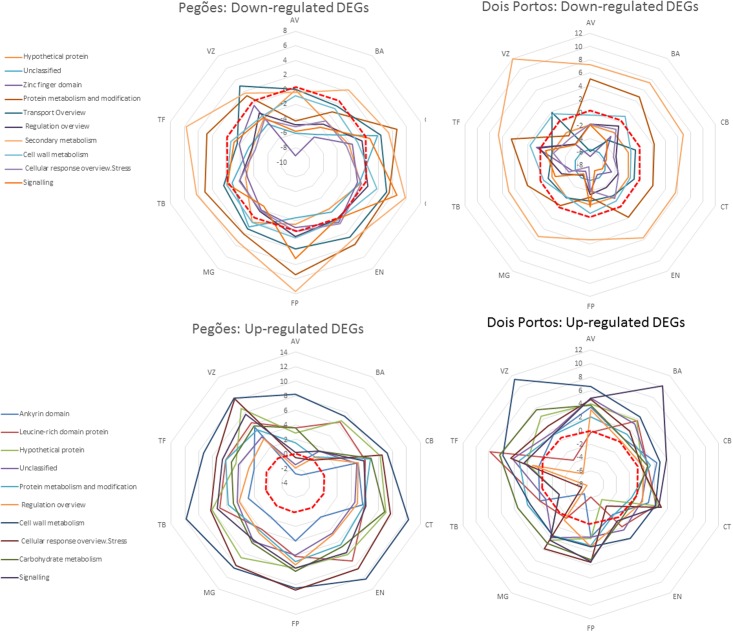
Relative relevance [average log_2(*gene expression ratio*)_] of functional categories of downregulated and upregulated DEGs in the varieties analyzed in Dois Portos and Pegões, by MapMan analysis.

When comparing the relevance of the identified functional categories in each variety in both locations (**Figure 4**) it is possible to see that, in Pegões, many downregulated categories were upregulated in all varieties, such as secondary metabolism, protein metabolism and transport while most upregulated categories remained upregulated. On the contrary, upregulated DEGs followed the expected pattern in all varieties except AV, that had four bins downregulated. In Dois Portos the upregulation of downregulated secondary metabolism DEGs was common to all varieties while protein metabolism was upregulated in eight out of 12 varieties. Surprisingly, TB showed a significant downregulation of upregulated stress response and regulation DEGs, while overall, upregulated DEGs followed the expected pattern in the other varieties.

### Tolerance and Sensitivity of the Varieties

**FIGURE 5:**
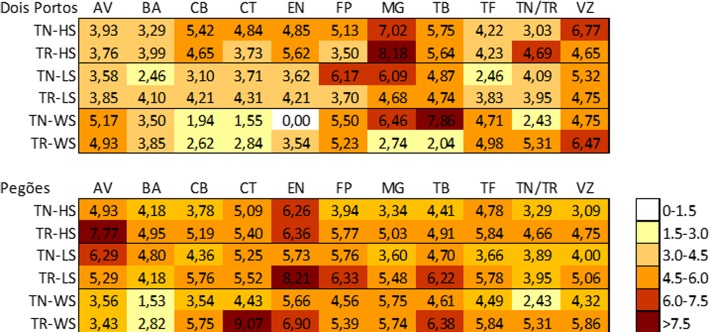
*Stress Matrix* built using the average of the |log_2(*gene expression ratio*)_| of HS/LS/WS DEGs that yielded a significant value (|>1.5|) of the expected regulation (down-/up-).

In both locations, varieties were ranked as sensitive/tolerant to abiotic stress in the field and relative to the individual stresses (HS, LS, and WS) tested; using the values of expression of the chosen up and downregulated DEGS that were associated with each stress (Supplementary Table S2). The results obtained were compared with those of TN (reference tolerant variety) and TR (reference sensitive variety) in the above-mentioned stress conditions. Thus, a stress matrix was obtained (**Figure 5**). In Pegões, the varieties were subjected to higher levels of stress, consistent with the geographic locations and the meteorological data of the areas. AV, EN, and TB were the varieties which suffered the most and all the stresses were equally relevant. In Dois Portos, MG, TB, and VZ were the most affected varieties while BA and CT suffered the least. In this location heat and drought were the most forceful stresses.

**FIGURE 6:**
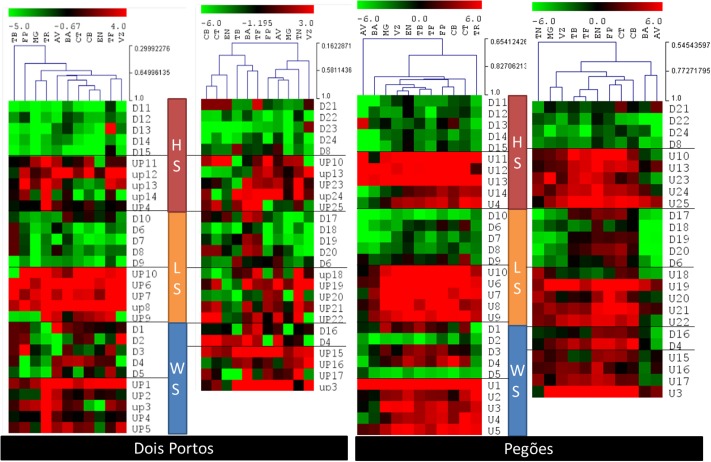
Relative gene expression ratios obtained by RT-qPCR of the 65 DEGs quantified in the twelve varieties in both locations (Dois Portos and Pegões). Relative expressions in relation to control plants of TR (left) and TN (right) were calculated and values were normalized with respect to *translation initiation factor eIF-3 subunit 4* (*TIF*), *translation initiation factor eIF-2B alpha subunit* (*TIF-GTP*) and *actin 2* (*act*) mRNA. The data correspond to log_2(*gene expression ratio*)_ of three independent samples measured in duplicate.

In Pegões, the individual expression of the array of stress DEGs correlated more closely with the expected patterns, with the exception of downregulated TN-drought DEGs, that were upregulated in all varieties except AV (**Figure 6**). Heat and light DEGs, individually, showed the expected expression patterns as expected and were able to cluster the varieties together, according to their levels of expression. The varieties with a pattern of expression more distant from TN and TR are AV and BA, while VZ and MG correlated closely with TN and CT and CB with TR. In Dois Portos, as environmental conditions were milder, the differences aren’t as stark, especially when comparing with TN. Upregulated and downregulated light DEGs and downregulated heat DEGs yielded the expected regulation patterns.

From the combined analysis of the matrix and the individual expression of the stress DEGs it is already possible to characterize tolerance/sensitivity and to identify specific patterns of response to each stress. It is thus possible to see that in AV there was no clear pattern in Pegões but in Dois Portos it behaved as sensitive to light and was overall tolerant to field stress. In Dois Portos BA was tolerant to light and drought and in Pegões it was tolerant to heat and drought. The genotype of CT used in Dois Portos showed tolerance to heat and drought and the one used in Pegões was sensitive to all three individual stresses and also to field stress.

‘Cerceal Branco’ (CB) in Pegões was sensitive to heat but tolerant to light and drought, also showing indications of overall tolerance in the field. In Dois Portos it was tolerant to light and drought, which is in accordance with its previous description. In Dois Portos EN was tolerant to the three individual abiotic stresses, as previously described (IVV, 2011) and in Pegões it showed sensitivity to heat and light, confirming the empirical opinion on this variety (Martins and Gonçalves, personal communication).

‘Fernão Pires’ was sensitive to light and drought in Dois Portos and in Pegões to heat and to field conditions of stress, as described before (IVV, 2011). ‘MG’ was sensitive to heat, light, drought, and field stress in Dois Portos and in Pegões it was sensitive to drought, which goes against its described characterization (IVV, 2011).

‘Tinta Barroca’ was identified as sensitive to light in Dois Portos and Pegões. ‘TF in Dois Portos was sensitive to heat and drought and tolerant to high light and in Pegões it was tolerant to heat and light and sensitive to drought. ‘Viozinho’ (VZ) was tolerant to light in Dois Portos and in Pegões it showed tolerance to heat. It also showed tolerance to field stress.

### Custom Array

The custom array should comprise 48 genes in total, including the three best reference genes for abiotic stress in grapevine (Coito et al., 2012). Of the 65 DEGs used in this work, some proved to be better markers of specific stresses than others. To obtain the 45 best representatives of each stress condition, significantly expressed DEGs were identified. All those that did not yield the expected result in more than half of the varieties were discarded (code shaded in gray in Supplementary Table S1). It is possible to see that the number of upregulated DEGs discarded was evenly distributed between stresses, with only a slightly higher number on WS. As to downregulated DEGs, again WS had more DEGs discarded than the other stresses.

## Discussion

Several custom-made RT-qPCR arrays are available in the market, for a number of organisms such as human, mouse, rat, and zebrafish and they usually comprise primers for cancer related, immunopathology related, development related and differentiation related genes (TaqMan^®^ Gene Expression Assays, from Life Technologies^3^, SureFIND^TM^ Transcriptome PCR Arrays from Qiagen’s SABiosciences division^4^ and KiCqStart^®^ SYBR^®^ Green Primers from Sigma-Aldrich^5^). These arrays are flexible, expedite and time-saving offers for targeted research. Unfortunately there are no such products available for research in any plant species, despite their complexity and high economic value. In this work, a custom-made RT-qPCR array to assess the sensitivity of traditional Portuguese grapevine varieties to abiotic stresses was elaborated based on previously obtained data from well-studied varieties (Rocheta et al., 2016), that were studied in imposed abiotic stress treatments (drought, heat, and excess light) under controlled conditions and also in the field. Several DEGs identified in this previous work were tested in 10 traditional varieties on two different locations with distinct climate patterns. An attempt was also made at trying to assess the most forceful of those abiotic stresses affecting each variety in each field location. Finally, 45 DEGs were chosen as consistent stress markers and were thus included in the custom array.

Traditional and internationally recognized wine regions have exclusive *terroirs*, a concept associated with the environmental and cultural landscapes that are an important component of local economies, through production, processing, trade, and enotourism (Jones et al., 2005). To prepare for impending changes in those regions, the wine industry must adjust to climate change and focused research is needed in identifying and cataloging varietal potential, phenological changes due to climate changes and wine quality.

However, most of the characteristics of grapevine that have the greatest economic relevance (such as yield and quality of the must) are traits with continuous and symmetric distributions (i.e., statistically normal distributions) in heterogeneous populations and are termed “quantitative traits” (Gonçalves and Martins, 2012). These traits are determined by a large number of genes with low and cumulative actions and have strong environmental deviations. Tolerance to stress also falls within this category of trait and that is the reason why tolerance to stress can vary within an ancient variety up to several fold, and that explains why some varieties show very distinct patterns of response to stress (e.g., TN and TR; Carvalho et al., 2015, 2016). Nowadays, all this variability is useful for the practical purposes of identifying varieties (and genotypes within varieties) that are tolerant to abiotic stress and therefore that can withstand climate changes without major changes in yield and quality.

### The Application of a Custom qPCR Abiotic Stress Array

It is widely accepted that the grapevine transcriptome is very plastic and easily changes with environmental conditions, especially from season to season (Dal Santo et al., 2013). Nevertheless, it is possible to pinpoint a number of DEGs that may follow the same pattern in different genotypes cultivated in distinct environments. The DEGs tested for this custom array comprised genes differentially expressed in two grapevine varieties with contrasting patterns of response to stress (Carvalho et al., 2015, 2016; Rocheta et al., 2016). These DEGs belong to the most representative functional categories present in MapMan ontology (Thimm et al., 2004), comprising files specifically designed for grapevine (Pontin et al., 2010). Twenty of these DEGs were discarded, as they did not yield the expected expression tendency in most varieties. This result, globally, is not unexpected, as the chosen DEGs were already identified as variety-specific (Rocheta et al., 2016). However, some individual results are indeed unexpected, as is the case of the dismissal of *LEA D-29* and *HSP26*.*5* from the DEGs assigned as upregulated under WS. These genes are described as upregulated by drought in rice and cotton (Xiao et al., 2007; Venkata et al., 2012) and were also upregulated in TR but not in TN (Rocheta et al., 2016). Here, in Dois Portos they were upregulated only in AV, an indication that they may be variety-specific and thus not appropriate for a wide-use array. Other WS upregulated DEGs discarded were *dehydrin 1* and *protein phosphatase 2C*, the first widely reported as a key factor in drought response (Xiao and Nassuth, 2006) while the second may actually impair the efficiency of response to drought (Liu et al., 2009). Light stress upregulated DEGs discarded were two *proline-rich proteins*, both indirectly related to the response to high light (Zhan et al., 2012) and therefore probably consisting in a variety-specific response associated only to TN, CB, CT, and MG in Pegões. Surprisingly, *HSP18.2* was removed from the final list of HS DEGs, as none of the 10 varieties studied in Dois Portos showed upregulation of this DEG. In this specific case, it can be considered that the response of this gene was more region-specific, as the level of heat stress in Dois Portos is much lower than it is in Pegões, where most of the varieties presented upregulation of this gene. Nevertheless, as the custom array is built to be used in different climate conditions, this DEG was removed.

Previous studies of abiotic stress response in the grapevine variety Aragonez assigned metabolism and its sub-bin protein folding as the mostly upregulated bin in HS and WS (Rocheta et al., 2014). In our study, this bin (protein metabolism and modification) was well distributed between downregulated and upregulated DEGs but the overall tendency observed in most of the varieties studied here, both in Dois Portos and in Pegões, was indeed for a strong upregulation of DEGs belonging to this bin (even those previously identified as downregulated). In grapevine subjected to drought and salinity the most representative functional categories were also protein fate and metabolism (Cramer et al., 2007) as was the case of rice under drought (Gorantla et al., 2007). In *Quercus suber* subjected to drought, the most representative bins were metabolism, response to stimulus, and cellular processes (Magalhães et al., 2016), which falls in line with the behavior of all the grapevine varieties studied here, with the exception of AV in Pegões and TB in Dois Portos. In a recent study of several varieties subjected do defoliation, common markers of response to that treatment were found (Zenoni et al., 2017), an indication that, despite a high degree of varietal specificity there is some room for finding universal indicators to use as a screening method. Even varieties with different levels of tolerance to a specific stress, such as ‘Cabernet Sauvignon,’ ‘Riparia Gloire,’ and ‘Ramsey’ in relation to drought, the response of ABA and ethylene signaling hubs was found to be highly similar (Hopper et al., 2016). Nevertheless, if the differences in tolerance are related to a physiologically specific mechanism such as whether the variety in question is isohydric or anisohydric, the type of response is specific to each condition, with isohydric varieties showing faster transcriptome response, swifter ABA-related gene modulation, higher HSP expression levels and faster return to basal levels once the stress subsides (Dal Santo et al., 2016). Anisohydric varieties, on the other hand, show stronger activation of ROS-scavenging enzymes, molecular chaperones and abiotic stress-related genes (Dal Santo et al., 2016).

Heat promotes vegetative growth and hinders carbon balance, disturbing flower set and young berry development. Primary and secondary fruit metabolisms suffer changes, namely the delay of sugar and polyphenol accumulation during ripening (Torregrosa et al., 2017). High temperature is also reported to trigger the expression of genes linked to cell expansion in grape berries, impairing the expression of specific genes and delaying the onset of others (Rienth et al., 2016). In fact, the regulation of the response to temperature-related abiotic stress in berries is so tightly controlled that several genes, namely those associated with acidity and phenylpropanoid pathway suffer differential regulation whether the stress occurs at day or at night (Rienth et al., 2014).

These responses are also tissue-specific, even though transcriptomic studies in leaves when compared to berries show significant differences in the major regulated categories, with grapevine leaves under normal conditions showing increased levels of secondary metabolism transcripts (alkaloids, anthocyanins, diterpenoids, monoterpenoids, and flavonoids) (Pervaiz et al., 2016). Transcriptomic studies in berries show significantly high levels of genes related to cell wall metabolism, grape berry coloration, sugar/acid ratio but also genes related to oxidative stress response (Terrier et al., 2005; Rienth et al., 2016).

## Conclusion

As environmental changes in the Mediterranean area keep raising concern on the future of viticulture and irrigation is becoming more and more common, with all the related drawbacks (Howell, 2001; Liu et al., 2012) more efforts are required to understand the biology and physiology of such an economically significant species. This task is a strenuous one, as there are hundreds of different varieties, each comprising hundreds of different genotypes. Over the last years, novel tools have helped study several aspects of grapevine biology at the genetic and molecular levels, such as gene/protein characterization, population genomics, and transcriptomics. However, a thorough characterization of the response of varieties/genotypes to environmental stress is essential for the adaptation of viticulture to climate changes. A significant step will be accomplished with the development of a molecular tool to identify the level of tolerance/sensitivity to stress of a particular genotype and to which abiotic stress(es) it may be more adjusted. In the present report, we tested such a tool in leaves of 10 traditional Portuguese grapevine varieties, grown in two locations, with different levels of environmental constraints. We show that this tool can be used to identify a genotype’s tolerance to stress and therefore its adaptability to withstand environmental stress. Therefore, the present report opens new research and strategic possibilities, as this tool can be commercially customed and used to characterize genotypes with yet unknown behavior upon stress.

## Author Contributions

LC and SA designed the experiments and contributed to the data interpretation. LC and MS undertook the experiment and collected the data. LC analyzed the data and drafted the manuscript, together with SA. JC and MR contributed with results in TN and TR. All authors contributed to the revision of the manuscript and approved the final manuscript.

## Conflict of Interest Statement

The authors declare that the research was conducted in the absence of any commercial or financial relationships that could be construed as a potential conflict of interest.

## Footnotes

1 https://www.ipma.pt/pt/

2 http://www.ncbi.nlm.nih.gov/

3 https://www.thermofisher.com/us/en/home/life-science/pcr/real-time-pcr/real-time-pcr-assays/taqman-gene-expression.html

4 http://www.sabiosciences.com/transcriptomepcrarray.php

5 http://www.sigmaaldrich.com/catalog/product/sigma/kspq12012?lang=pt&region=PT
