# Supplementary material for: Design of a Custom RT-qPCR Array for Assignment of Abiotic Stress Tolerance in Traditional Portuguese Grapevine Varieties
Source: Front Plant Sci. 2017 Oct 25;8:1835. doi: 10.3389/fpls.2017.01835 (PMC5660995; doi:10.3389/fpls.2017.01835)
Supplement: Supplementary file 1 [file Table_1.DOCX]

| **Table S1│** Characterization of the field conditions and of the controlled growth-room stress treatments applied to the control varieties and description of field conditions where the ten traditional varieties were grown. | | | | | | |
| --- | --- | --- | --- | --- | --- | --- |
|  | **Control varieties** | | | | | |
|  | Field (Pegões) | Growthroom controlled conditions | | | | |
|  |  | Control | | HS | WS | LS |
| TN  TR | Location/soil/climate/rootstock: see below;  Irrigation:  FI (Fully irrigated), applied with drip emitters (4.0 L h^-1^) two per vine, 30 cm from the vine trunk, supplied according to evapotranspiration (ETc);  Ψw ≈ -0.2 MPa  NI (non irrigated), Ψw ≈ -0.7 MPa. | 3L pots;  200 µmol m^-2^ s^-1^ irradiance, 16h light/8h dark photoperiod,  25 ºC day/ 23 ºC night; well-watered with nutrient solution | | one hour at 42 ºC (provided by a homogenous heat source measured at the surface of the leaf) | watering withdrawn *(circa* 4 days)  Ψw ≈ -0.9 MPa (severe water stress, Chaves *et al*., 2002) | one hour at 2,000 µmol·m^-2^·s^-1^ |
| **Traditional Varieties** | | | | | | |
| Pegões | | | Dois Portos | | | |
| AV BA CB CT EN FP MG TB TF VZ | Location: 38º 38’ 55” N; -8º 39’ 14” O; Climate: Mediterranean with hot dry summers and mild winters;  Soil: no slope, drainage conditions good. The soil is originated from podzols, sandy surface layer, clay is reached at 1.0 m depth.  Rootstock: 1103 Paulsen.  Irrigation: NI (non irrigated), Ψw = -0.7 + MPa | | Location: 38º 81’ 67.85” N; -9º 14’ 60.78” O; Climate: Atlantic, with mild summers with some humidity and cool winters;  soil: no slope, drainage conditions good. The soil is derived from alluvium of calcareous and sandstone rocks from Jurassic and Cretacic origin, water at 4 m depth.  Rootstock: SO4  Irrigation: NI (non irrigated), Ψw = no data available | | | |
